# Body mass index and risk of progression from monoclonal gammopathy of undetermined significance to multiple myeloma: Results from the Prostate, Lung, Colorectal and Ovarian Cancer Screening Trial

**DOI:** 10.1038/s41408-022-00642-4

**Published:** 2022-04-01

**Authors:** Vicky C. Chang, Ali A. Khan, Wen-Yi Huang, Hormuzd A. Katki, Mark P. Purdue, Ola Landgren, Jonathan N. Hofmann

**Affiliations:** 1 Division of Cancer Epidemiology and Genetics, National Cancer Institute, Bethesda, MD USA; 2 Myeloma Program, Sylvester Comprehensive Cancer Center, University of Miami, Miami, FL USA

**Keywords:** Epidemiology, Risk factors, Myeloma, Cancer epidemiology

## Dear Editor,

Monoclonal gammopathy of undetermined significance (MGUS) is an asymptomatic precursor condition that precedes nearly all cases of multiple myeloma (MM) [1] and is typically characterized by the presence of a monoclonal (M)-protein in serum [2]. MGUS affects approximately 2–3% of the general US population 50 years of age or older [3, 4] and has an estimated annual average risk of progression of 1% [5]. Obesity (and high body mass index [BMI]) is increasingly recognized as an established risk factor for MM, in addition to advancing age, male sex, Black race, and genetic susceptibility [6]. Limited and inconsistent evidence exists to support an association of obesity or BMI with MGUS [7], suggesting that excess body fatness may play a more important role in later stages of MM development. However, it remains unclear whether high BMI or other modifiable lifestyle factors are associated with progression from MGUS to MM, especially after controlling for established clinical risk factors for progression (e.g., immunoglobulin isotype, M-protein concentration, free light-chain [FLC] ratio, immunoparesis) [5, 8].

In the current issue of *Blood Cancer Journal*, Kleinstern et al. [9] investigated the relationship between BMI and progression from MGUS to MM and other plasma-cell or lymphoid disorders among 594 individuals identified to have MGUS from a population-based MGUS screening study in Olmsted County, Minnesota. The authors found a suggestive association between high BMI, assessed close to the time of screening (within 2 years for 80% of subjects), and increased risk of MGUS progression independent of clinically established risk factors for progression, with a stronger association observed among females than males. To our knowledge, Kleinstern et al. [9] were the first to examine potential sex differences in BMI and MGUS progression; previous studies consisted almost entirely of males [10] or had limited sample size to perform stratified analyses [11, 12]. To further address this question, we were motivated to conduct a study within the Prostate, Lung, Colorectal and Ovarian (PLCO) Cancer Screening Trial to evaluate the association between BMI and risk of progression from MGUS to MM, overall and by sex.

The study population for this investigation was drawn from participants 55–74 years of age who were randomized to the screening arm of the PLCO Cancer Screening Trial between 1993 and 2001. As described previously [8], MGUS was characterized in prediagnostic sera from selected participants who did and did not subsequently develop MM or other hematologic malignancies during follow-up. For the current investigation, we restricted our analyses to participants with non-IgM MGUS (*n* = 488, including 324 non-progressing MGUS and 164 MM); IgM MGUS was excluded because it typically progresses to Waldenstrom macroglobulinemia rather than MM [2]. As a secondary analysis, we also included another 246 participants with light-chain (LC)-MGUS (*n* = 216 non-progressing LC-MGUS and 30 LC-MM).

BMI was calculated based on height and weight reported by participants in the baseline PLCO questionnaire. Given the nested case-control study design and consistent with previous investigations within this study population [8, 13], we used multivariable logistic regression to estimate odds ratios (OR) and 95% confidence intervals (CI) for the associations between BMI (modeled as either a categorical or continuous variable) and risk of progression from non-IgM MGUS to MM or LC-MGUS to LC-MM. All basic models adjusted for demographic and study design-related factors, including sex (overall model only), age at blood draw, race, study center, and year of blood draw. Full models additionally adjusted for MGUS characteristics previously associated with progression (immunoglobulin isotype, M-protein concentration, serum FLC ratio, and immunoparesis), to evaluate whether BMI is associated with MGUS progression independent of these established clinical risk factors [5, 8]. Participants with missing data on BMI or any covariate (*n* = 9) were excluded, leaving a final analytic dataset of 482 with non-IgM MGUS and 243 with LC-MGUS. Analyses were conducted for both sexes combined and stratified by sex.

The majority of participants in this investigation were males and non-Hispanic white (demographic and clinical characteristics, overall and stratified by sex, presented for non-IgM MGUS/MM and LC-MGUS/LC-MM in Supplementary Tables 1 and 2, respectively). All female participants were postmenopausal at baseline, except for two (1%) with unknown menopausal status. As expected, compared with non-progressors, participants with non-IgM MGUS that progressed to MM were more likely to have IgA isotype (29% vs. 15%), elevated M-protein concentration (38% vs. 3%), abnormal serum FLC ratio (77% vs. 36%), and presence of immunoparesis (i.e., suppression of at least one uninvolved immunoglobulin; 57% vs. 19%) (*P* < 0.05 for all clinical risk factors, overall and among both sexes).

Table 1 presents associations between BMI and progression from non-IgM MGUS to MM. Overall, each 5 kg/m^2^ increase in BMI was associated with a statistically significant 35% increase in odds of progression from non-IgM MGUS to MM (OR 1.35, 95% CI 1.03–1.77), independent of demographic and clinical characteristics. We also observed an elevated odds of progression for obese individuals, particularly those with class 2/3 obesity (≥35 vs. <25 kg/m^2^; OR 2.04, 95% CI 0.72–5.83). Sex-stratified analyses revealed a stronger association among females (per 5 kg/m^2^; OR 1.51, 95% CI 0.97–2.34) than males (OR 1.29, 95% CI 0.89–1.87); however, no statistically significant interaction was detected (*P*_interaction_ = 0.61). In addition, despite lack of statistical significance, we observed a nearly 3-fold increase in odds of progression for females who were obese (≥30 kg/m^2^; OR 2.90), whereas among males the odds of progression were only modestly increased for those in the class 2/3 obesity category (≥35 kg/m^2^; OR 1.50). Analyses excluding underweight participants (<18.5 kg/m^2^; *n* = 1 male and 3 females) yielded similar results (data not shown).

**Table 1:** Associations between BMI and risk of progression from non-IgM MGUS to MM, overall and stratified by sex.

BMI	*N*_MGUS_^a^	*N*_MM_^b^	Basic model^c^ OR (95% CI)	Full model^d^ OR (95% CI)
*Overall*				
<25 kg/m^2^	98	54	1.00 (Reference)	1.00 (Reference)
25–29.9 kg/m^2^	145	71	1.01 (0.61–1.66)	1.03 (0.55–1.92)
≥30 kg/m^2^	76	38	1.29 (0.71–2.32)	1.24 (0.59–2.60)
30–34.9 kg/m^2^	52	25	1.22 (0.63–2.38)	0.93 (0.39–2.22)
≥35 kg/m^2^	24	13	1.44 (0.60–3.47)	2.04 (0.72–5.83)
Per 5 kg/m^2^ increase	319	163	1.25 (1.00–1.55)	1.35 (1.03–1.77)
*Males*				
<25 kg/m^2^	60	32	1.00 (Reference)	1.00 (Reference)
25–29.9 kg/m^2^	110	56	1.02 (0.55–1.87)	0.90 (0.41–1.97)
≥30 kg/m^2^	57	22	0.92 (0.43–1.94)	0.91 (0.35–2.36)
30–34.9 kg/m^2^	42	18	1.01 (0.46–2.25)	0.77 (0.27–2.19)
≥35 kg/m^2^	15	4	0.63 (0.16–2.43)	1.50 (0.33–6.76)
Per 5 kg/m^2^ increase^e^	227	110	1.09 (0.81–1.48)	1.29 (0.89–1.87)
*Females*				
<25 kg/m^2^	38	22	1.00 (Reference)	1.00 (Reference)
25–29.9 kg/m^2^	35	15	0.97 (0.37–2.53)	1.30 (0.35–4.77)
≥30 kg/m^2^	19	16	2.53 (0.85–7.50)	2.90 (0.73–11.5)
30–34.9 kg/m^2^	10	7	1.43 (0.36–5.72)	2.03 (0.32–12.9)
≥35 kg/m^2^	9	9	4.21 (1.09–16.2)	3.71 (0.74–18.7)
Per 5 kg/m^2^ increase^e^	92	53	1.52 (1.05–2.19)	1.51 (0.97–2.34)

*BMI* body mass index*, CI* confidence interval, *MGUS* monoclonal gammopathy of undetermined significance, *MM* multiple myeloma, *OR* odds ratio.

Note: Two sets of analyses were performed when BMI was examined as a categorical variable, including one with three BMI categories (<25, 25–29.9, and ≥30 kg/m^2^) and the other further dividing the obesity category (≥30 kg/m^2^) into two subcategories (results shown for 30–34.9 and ≥35 kg/m^2^).

^a^Number of participants with non-IgM MGUS that did not progress to MM.

^b^Number of participants with non-IgM MGUS that progressed to MM.

^c^Adjusted for sex (overall model only), age, age^2^, race (non-Hispanic white, non-Hispanic Black, other [Hispanic, Asian, and Pacific Islander]), study center (Upper Midwest [Wisconsin and Minnesota], West/South [Colorado, Hawaii, Missouri, Utah, and Alabama], East [Georgetown, Detroit, and Pittsburgh]), and calendar year of blood draw (1995–2000, 2001–2002, 2003–2006).

^d^Adjusted for variables in the basic model and additionally for immunoglobulin isotype (IgA, IgG, biclonal), elevated M-protein concentration (no, yes [≥15 g/L]), serum free light-chain ratio (normal [0.26–1.65], abnormal [<0.26 or >1.65]), and immunoparesis (number of uninvolved immunoglobulins below the lower level of the normal reference range; none, 1, 2, biclonal).

^e^*P* for multiplicative interaction between sex and continuous BMI = 0.15 (basic model) and 0.61 (full model).

In our secondary analysis examining BMI and progression from LC-MGUS to LC-MM (Supplementary Table 3), associations appeared to be in the positive direction for females (per 5 kg/m^2^; OR 1.27) but not males (OR 0.59) or both sexes combined (OR 0.82); however, these results should be interpreted with caution owing to the small number of LC-MM cases. Furthermore, in analyses combining non-IgM MGUS and LC-MGUS (Supplementary Table 4), higher BMI was associated with significantly increased odds of progression in females (per 5 kg/m^2^; OR 1.48, 95% CI 1.03–2.13) but not in males or both sexes combined.

In this case-control study within the prospective PLCO Cancer Screening Trial, we found that higher baseline BMI was associated with an increased future risk of progression from non-IgM MGUS to MM, independent of clinical characteristics of MGUS, and that this association was more prominent among females than males. Notably, despite lack of statistical significance, our results suggest that obese females with MGUS may have a nearly three-fold increased risk of progression compared to those who are normal or underweight. Our findings, including sex-specific results, are consistent with those reported by Kleinstern et al. [9], although their study also included IgM MGUS and progression to hematologic diseases other than MM.

Prior to the investigation by Kleinstern et al. [9], two other prospective studies observed positive associations between obesity (or high BMI) and progression from MGUS to MM, including an analysis of administrative health data from a large cohort of US veterans [10] and a population-based screening study in Iceland [11]. However, these studies were limited by the lack of information on clinical characteristics of MGUS or had limited case numbers to examine MGUS subtypes or sex-specific associations. To our knowledge, our study is the first to simultaneously examine associations between BMI and progression from both non-IgM MGUS to MM and LC-MGUS to LC-MM. Beyond the observed association of high BMI with progression from non-IgM MGUS to MM overall and in females, we also observed a suggestive association with progression from LC-MGUS to LC-MM in females only; however, our results for LC-MGUS/LC-MM warrant additional investigation in larger samples. A possible mechanism through which higher BMI may contribute to MGUS progression may involve adiponectin, a hormone with anti-inflammatory and insulin-sensitizing properties and known to be under-expressed in obese individuals [14]. Specifically, previous studies have reported lower levels of circulating adiponectin among MM patients compared to those with non-progressing MGUS, supporting the potential role of obesity in MGUS progression [15, 16].

Similar to Kleinstern et al. [9], our finding of a stronger association between BMI/obesity and MGUS progression to MM among females is intriguing and requires confirmation in future studies. Although underlying mechanisms remain to be elucidated, differences in endogenous sex hormones, such as estrogen, may provide a plausible explanation for this potential sex difference. For instance, estrogen is known to be elevated in postmenopausal obese women and has been shown to promote the progression of MM through immunosuppressive pathways in a female mouse model [17]. Furthermore, sex differences in bone marrow adipose tissue have been reported previously, with older (postmenopausal) females having higher vertebral marrow fat content compared to older males [18], and may be another possible mechanism underlying the stronger association we observed between high BMI and MGUS progression in females. Interestingly, one prospective study noted an inverse association between serum adiponectin levels and progression from MGUS to MM among females but not males [15], whereas a larger cross-sectional study reported no sex differences in this association [16].

Strengths of our study included availability of prediagnostic information on BMI and MGUS status within a prospective cohort, detailed assessment of and adjustment for clinical characteristics previously associated with MGUS progression, and the ability to evaluate—to our knowledge, for the first time—the associations between BMI and progression to MM according to specific MGUS subtypes (i.e., non-IgM and LC-MGUS). Our study also had several limitations, including self-reported information on BMI and a relatively small sample size that limited statistical power, especially for sex-stratified analyses of LC-MGUS/LC-MM. Future research is warranted in larger studies to investigate the possibility of incorporating BMI (and related biomarkers) into absolute risk prediction models for MGUS progression, such as with competing time-to-event analyses.

In summary, our study contributes to growing evidence suggesting that high BMI or obesity may be a risk factor for progression from MGUS to MM, particularly among females. Given limited knowledge regarding modifiable factors associated with MGUS progression, these findings, if replicated in larger future studies, may have implications for clinical management and risk prediction and stratification among MGUS patients.

## Supplementary information

Supplementary Tables 1–4
