# Supplementary material for: Body mass index and risk of progression from monoclonal gammopathy of undetermined significance to multiple myeloma: Results from the Prostate, Lung, Colorectal and Ovarian Cancer Screening Trial
Source: Blood Cancer J. 2022 Apr 1;12(4):51. doi: 10.1038/s41408-022-00642-4 (PMC8975932; doi:10.1038/s41408-022-00642-4)
Supplement: Supplementary file 1 — Supplementary Tables 1–4 [file 41408_2022_642_MOESM1_ESM.pdf]

Supplementary Table 1. Demographic and clinical characteristics of study participants with non-IgM MGUS that progressed or did not progress to MM, overall and stratified by sex

Characteristic	Overall: Non-progressing MGUS (*N* = 324)	Overall: Progressing MGUS to MM (*N* = 164)	Males: Non-progressing MGUS (*N* = 228)	Males: Progressing MGUS to MM (*N* = 111)	Females: Non-progressing MGUS (*N* = 96)	Females: Progressing MGUS to MM (*N* = 53)
Sex, *n* (%)						
Male	228 (70.4)	111 (67.7)				
Female	96 (29.6)	53 (32.3)				
Age at blood draw[a], years, mean (SD)	70.3 (5.9)	67.9 (5.1)	70.3 (5.8)	67.5 (5.1)	70.5 (6.2)	68.8 (5.1)
Race[b], *n* (%)						
Non-Hispanic white	215 (66.4)	148 (90.2)	157 (68.9)	102 (91.9)	58 (60.4)	46 (86.8)
Non-Hispanic Black	79 (24.4)	10 (6.1)	51 (22.4)	4 (3.6)	28 (29.2)	6 (11.3)
Other[c]	30 (9.3)	6 (3.7)	20 (8.8)	5 (4.5)	10 (10.4)	1 (1.9)
Study center, *n* (%)						
Upper Midwest	137 (42.3)	59 (36.0)	104 (45.6)	47 (42.3)	33 (34.4)	12 (22.6)
West/South	93 (28.7)	62 (37.8)	63 (27.6)	35 (31.5)	30 (31.3)	27 (50.9)
East	94 (29.0)	43 (26.2)	61 (26.8)	29 (26.1)	33 (34.4)	14 (26.4)
Calendar year of blood draw, *n* (%)						
1995–2000	88 (27.2)	69 (42.1)	63 (27.6)	54 (48.6)	25 (26.0)	15 (28.3)
2001–2002	125 (38.6)	64 (39.0)	90 (39.5)	42 (37.8)	35 (36.5)	22 (41.5)
2003–2006	111 (34.3)	31 (18.9)	75 (32.9)	15 (13.5)	36 (37.5)	16 (30.2)
BMI, kg/m^2, mean (SD)	27.5 (4.8)	27.9 (5.3)	27.8 (4.6)	27.6 (4.3)	26.7 (5.1)	28.3 (6.9)
BMI categories, *n* (%)						
<18.5	4 (1.3)	0 (0)	1 (0.4)	0 (0)	3 (3.3)	0 (0)
18.5–24.9	95 (29.7)	54 (33.1)	60 (26.3)	32 (29.1)	35 (38.0)	22 (41.5)
25–29.9	145 (45.3)	71 (43.6)	110 (48.2)	56 (50.9)	35 (38.0)	15 (28.3)
30–34.9	52 (16.3)	25 (15.3)	42 (18.4)	18 (16.4)	10 (10.9)	7 (13.2)
≥35	24 (7.5)	13 (8.0)	15 (6.6)	4 (3.6)	9 (9.8)	9 (17.0)
Missing	4	1	0	1	4	0
Immunoglobulin isotype, *n* (%)						
IgG	256 (79.0)	112 (68.3)	179 (78.5)	77 (69.4)	77 (80.2)	35 (66.0)
IgA	50 (15.4)	47 (28.7)	39 (17.1)	32 (28.8)	11 (11.5)	15 (28.3)
Biclonal	18 (5.6)	5 (3.0)	10 (4.4)	2 (1.8)	8 (8.3)	3 (5.7)
Elevated M-protein concentration, *n* (%)						
No	312 (96.6)	101 (61.6)	220 (96.9)	65 (58.6)	92 (95.8)	36 (67.9)
Yes (≥15 g/L)	11 (3.4)	63 (38.4)	7 (3.1)	46 (41.4)	4 (4.2)	17 (32.1)
Missing	1	0	1	0	0	0
Serum FLC ratio[d], *n* (%)						
Normal (0.26–1.65)	207 (63.9)	38 (23.2)	141 (61.8)	27 (24.3)	66 (68.8)	11 (20.8)
Abnormal (<0.26 or >1.65)	117 (36.1)	126 (76.8)	87 (38.2)	84 (75.7)	30 (31.3)	42 (79.2)
Immunoparesis[e] (excluding biclonal MGUS), *n* (%)						
None	247 (80.7)	68 (42.8)	181 (83.0)	49 (45.0)	66 (75.0)	19 (38.0)
1	51 (16.7)	46 (28.9)	32 (14.7)	26 (23.9)	19 (21.6)	20 (40.0)
2	8 (2.6)	45 (28.3)	5 (2.3)	34 (31.2)	3 (3.4)	11 (22.0)

BMI body mass index, *FLC* free light-chain, *MGUS* monoclonal gammopathy of undetermined significance, *MM* multiple myeloma, *SD* standard deviation.

[a]Age at the most recent blood draw prior to diagnosis or selection.

[b]Participants who were Black or Asian/Pacific Islander were oversampled for the MGUS screening effort in the PLCO Cancer Screening Trial.

[c]Including Hispanic, Asian, and Pacific Islander.

[d]Ratio of serum kappa to lambda free light-chain levels.

[e]Number of uninvolved immunoglobulins below the lower level of the normal reference range (IgG MGUS: IgA and/or IgM suppressed; IgA MGUS: IgG and/or IgM suppressed).

Supplementary Table 2. Demographic and clinical characteristics of study participants with LC-MGUS that progressed or did not progress to LC-MM, overall and stratified by sex

	Overall		Males		Females	
**Characteristic**	**Non-progressing LC-MGUS (*N* = 216)**	**Progressing LC-MGUS to LC-MM (*N* = 30)**	**Non-progressing LC-MGUS (*N* = 144)**	**Progressing LC-MGUS to LC-MM (*N* = 12)**	**Non-progressing LC-MGUS (*N* = 72)**	**Progressing LC-MGUS to LC-MM (*N* = 18)**
Sex, *n* (%)						
Male	144 (66.7)	12 (40.0)				
Female	72 (33.3)	18 (60.0)				
Age at blood draw[a], years, mean (SD)	70.8 (5.5)	67.2 (5.7)	71.1 (5.4)	68.5 (5.5)	70.1 (5.7)	66.4 (5.8)
Race[b], *n* (%)						
Non-Hispanic white	133 (61.6)	29 (96.7)	96 (66.7)	11 (91.7)	37 (51.4)	18 (100)
Non-Hispanic Black	79 (36.6)	0 (0)	45 (31.3)	0 (0)	34 (47.2)	0 (0)
Other[c]	4 (1.9)	1 (3.3)	3 (2.1)	1 (8.3)	1 (1.4)	0 (0)
Study center, *n* (%)						
Upper Midwest	92 (42.6)	12 (40.0)	62 (43.1)	7 (58.3)	30 (41.7)	5 (27.8)
West/South	54 (25.0)	10 (33.3)	41 (28.5)	1 (8.3)	13 (18.1)	9 (50.0)
East	70 (32.4)	8 (26.7)	41 (28.5)	4 (33.3)	29 (40.3)	4 (22.2)
Calendar year of blood draw, *n* (%)						
1995–2000	63 (29.2)	13 (43.3)	48 (33.3)	7 (58.3)	15 (20.8)	6 (33.3)
2001–2002	94 (43.5)	10 (33.3)	55 (38.2)	2 (16.7)	39 (54.2)	8 (44.4)
2003–2006	59 (27.3)	7 (23.3)	41 (28.5)	3 (25.0)	18 (25.0)	4 (22.2)
BMI, kg/m^2, mean (SD)	28.0 (4.6)	27.0 (4.7)	28.0 (4.5)	26.7 (2.8)	28.0 (5.0)	27.2 (5.7)
BMI categories, *n* (%)						
<18.5	0 (0)	1 (3.3)	0 (0)	0 (0)	0 (0)	1 (5.6)
18.5–24.9	52 (24.4)	8 (26.7)	33 (23.4)	3 (25.0)	19 (26.4)	5 (27.8)
25–29.9	98 (46.0)	14 (46.7)	69 (48.9)	8 (66.7)	29 (40.3)	6 (33.3)
30–34.9	45 (21.1)	6 (20.0)	28 (19.9)	1 (8.3)	17 (23.6)	5 (27.8)
≥35	18 (8.5)	1 (3.3)	11 (7.8)	0 (0)	7 (9.7)	1 (5.6)
Missing	3	0	3	0	0	0
Immunoparesis[d], *n* (%)						
None	189 (87.5)	15 (50.0)	122 (84.7)	6 (50.0)	67 (93.1)	9 (50.0)
1	22 (10.2)	5 (16.7)	19 (13.2)	1 (8.3)	3 (4.2)	4 (22.2)
2 or 3	5 (2.3)	10 (33.3)	3 (2.1)	5 (41.7)	2 (2.8)	5 (27.8)

BMI body mass index, *LC* light-chain, *MGUS* monoclonal gammopathy of undetermined significance, *MM* multiple myeloma, *SD* standard deviation.
[a]Age at the most recent blood draw prior to diagnosis or selection.
[b]Participants who were Black or Asian/Pacific Islander were oversampled for the MGUS screening effort in the PLCO Cancer Screening Trial.
[c]Including Hispanic, Asian, and Pacific Islander.
[d]Number of immunoglobulins below the lower level of the normal reference range (IgG, IgA, and/or IgM suppressed).

Supplementary Table 3. Associations between BMI and risk of progression from LC-MGUS to LC-MM, overall and stratified by sex

**BMI**	$N_{\text{LC-MGUS}}$ [a]	$N_{\text{LC-MM}}$ [b]	**Basic model**[c] **OR (95% CI)**	**Full model**[d] **OR (95% CI)**
Overall				
<25 kg/m^2	52	9	1.00 (Reference)	1.00 (Reference)
25–29.9 kg/m^2	98	14	0.89 (0.30–2.65)	1.10 (0.34–3.57)
≥30 kg/m^2	63	7	0.54 (0.15–1.91)	0.60 (0.15–2.52)
Per 5 kg/m^2 increase	213	30	0.76 (0.47–1.25)	0.82 (0.47–1.41)
Males				
<25 kg/m^2	33	3	1.00 (Reference)	1.00 (Reference)
25–29.9 kg/m^2	69	8	1.22 (0.27–5.59)	1.50 (0.29–7.88)
≥30 kg/m^2	39	1	0.17 (0.01–2.44)	0.15 (0.01–2.97)
Per 5 kg/m^2 increase[e]	141	12	0.60 (0.24–1.53)	0.59 (0.21–1.62)
Females				
<25 kg/m^2	19	6	1.00 (Reference)	1.00 (Reference)
25–29.9 kg/m^2	29	6	1.21 (0.15–10.0)	2.05 (0.16–26.6)
≥30 kg/m^2	24	6	1.88 (0.24–14.9)	2.45 (0.20–29.8)
Per 5 kg/m^2 increase[e]	72	18	1.17 (0.56–2.44)	1.27 (0.54–3.01)

BMI body mass index, *CI* confidence interval, *LC* light-chain, *MGUS* monoclonal gammopathy of undetermined significance, *MM* multiple myeloma, *OR* odds ratio.

[a]Number of participants with LC-MGUS that did not progress to LC-MM.

[b]Number of participants with LC-MGUS that progressed to LC-MM.

[c]Adjusted for sex (overall model only), age, age^2, race (non-Hispanic white, other [non-Hispanic Black, Hispanic, Asian, and Pacific Islander]), study center (Upper Midwest [Wisconsin and Minnesota], West/South [Colorado, Hawaii, Missouri, Utah, and Alabama], East [Georgetown, Detroit, and Pittsburgh]), and calendar year of blood draw (1995–2000, 2001–2002, 2003–2006).

[d]Adjusted for variables in the basic model and additionally for immunoparesis (number of immunoglobulins below the lower level of the normal reference range; none, 1 or more).

[e]P for multiplicative interaction between sex and continuous BMI = 0.43 (basic model) and 0.39 (full model).

Supplementary Table 4. Associations between BMI and risk of progression from non-IgM MGUS or LC-MGUS to MM or LC-MM, overall and stratified by sex

BMI	N_{MGUS}[a]	N_{MM}[b]	**Basic model**[c] **OR (95% CI)**	**Full model**[d] **OR (95% CI)**
Overall				
<25 kg/m^2	150	63	1.00 (Reference)	1.00 (Reference)
25–29.9 kg/m^2	243	85	0.96 (0.62–1.49)	1.14 (0.65–1.98)
≥30 kg/m^2	139	45	1.01 (0.61–1.70)	1.14 (0.59–2.18)
30–34.9 kg/m^2	97	31	1.03 (0.58–1.82)	1.00 (0.48–2.08)
≥35 kg/m^2	42	14	0.99 (0.46–2.14)	1.47 (0.59–3.68)
Per 5 kg/m^2 increase	532	193	1.13 (0.93–1.37)	1.22 (0.97–1.54)
Males				
<25 kg/m^2	93	35	1.00 (Reference)	1.00 (Reference)
25–29.9 kg/m^2	179	64	1.00 (0.58–1.71)	1.03 (0.51–2.07)
≥30 kg/m^2	96	23	0.70 (0.36–1.39)	0.71 (0.30–1.68)
30–34.9 kg/m^2	70	19	0.79 (0.39–1.62)	0.62 (0.24–1.60)
≥35 kg/m^2	26	4	0.44 (0.13–1.54)	1.06 (0.27–4.17)
Per 5 kg/m^2 increase[e]	368	122	0.97 (0.74–1.27)	1.12 (0.80–1.55)
Females				
<25 kg/m^2	57	28	1.00 (Reference)	1.00 (Reference)
25–29.9 kg/m^2	64	21	0.97 (0.42–2.24)	1.51 (0.51–4.42)
≥30 kg/m^2	43	22	1.94 (0.81–4.65)	2.73 (0.92–8.08)
30–34.9 kg/m^2	27	12	1.47 (0.52–4.15)	2.50 (0.69–9.12)
≥35 kg/m^2	16	10	2.78 (0.90–8.59)	3.02 (0.78–11.7)
Per 5 kg/m^2 increase[e]	164	71	1.43 (1.05–1.94)	1.48 (1.03–2.13)

BMI body mass index, *CI* confidence interval, *LC* light-chain, *MGUS* monoclonal gammopathy of undetermined significance, *MM* multiple myeloma, *OR* odds ratio.

Note: Two sets of analyses were performed when BMI was examined as a categorical variable, including one with three BMI categories (<25, 25–29.9, and ≥30 kg/m^2) and the other further dividing the obesity category (≥30 kg/m^2) into two subcategories (results shown for 30–34.9 and ≥35 kg/m^2).

[a]Number of participants with non-IgM MGUS or LC-MGUS that did not progress to MM or LC-MM.

[b]Number of participants with non-IgM MGUS or LC-MGUS that progressed to MM or LC-MM.

[c]Adjusted for sex (overall model only), age, age^2, race (non-Hispanic white, non-Hispanic Black, other [Hispanic, Asian, and Pacific Islander]), study center (Upper Midwest [Wisconsin and Minnesota], West/South [Colorado, Hawaii, Missouri, Utah, and Alabama], East [Georgetown, Detroit, and Pittsburgh]), and calendar year of blood draw (1995–2000, 2001–2002, 2003–2006).

[d]Adjusted for variables in the basic model and additionally for immunoglobulin isotype (IgA, IgG, biclonal, LC-MGUS), elevated M-protein concentration (no, yes [≥15 g/L], LC-MGUS), serum free light-chain ratio (normal [0.26–1.65], abnormal [<0.26 or >1.65], LC-MGUS), and immunoparesis (number of uninvolved immunoglobulins below the lower level of the normal reference range; none, 1, 2 or 3, biclonal).

[e]*P* for multiplicative interaction between sex and continuous BMI = 0.13 (basic model) and 0.38 (full model).
